# “They have this not care – don’t care attitude:” A Mixed Methods Study Evaluating Community Readiness for Oral PrEP in Adolescent Girls and Young Women in a Rural Area of South Africa

**DOI:** 10.1186/s12981-020-00310-2

**Published:** 2020-09-07

**Authors:** Sarah E Nakasone, Natsayi Chimbindi, Nondumiso Mthiyane, Busisiwe Nkosi, Thembelihle Zuma, Kathy Baisley, Jaco Dreyer, Deenan Pillay, Sian Floyd, Isolde Birdthistle, Janet Seeley, Maryam Shahmanesh

**Affiliations:** 1 The University of Chicago, Chicago, USA; 2 Africa Health Research Institute, KwaZulu-Natal, South Africa; 3 Institute of Epidemiology and Health Care Mortimer Market Centre, University College London, London, WC1E 6JB UK; 4 London School of Hygiene & Tropical Medicine, London, UK

**Keywords:** Pre-exposure prophylaxis (PrEP), Adolescents, Health system readiness, Sexual and reproductive health services

## Abstract

**Introduction:**

Adolescent girls and young women (AGYW) remain disproportionately affected by HIV. In a rural area of South Africa with an annual incidence (2011–2015) of 5 and 7% per annum for 15–19 and 20–24-year olds respectively, oral pre-exposure prophylaxis (PrEP) could provide AGYW with a form of HIV prevention they can more easily control. Using quantitative and qualitative methods, we describe findings from a study conducted in 2017 that assessed knowledge of and attitudes toward PrEP to better understand community readiness for an AGYW PrEP rollout.

**Methods:**

We used descriptive analysis of a quantitative demographic survey (n = 8,414 ages 15–86) to identify population awareness and early PrEP adopters. We also conducted semi-structured, in-depth interviews with a purposive sample of 52 potential PrEP gatekeepers (health care workers, community leaders) to assess their potential influence in an AGYW PrEP rollout and describe the current sexual health landscape. Interviews were recorded, transcribed, and iteratively coded to identify major themes.

**Results:**

PrEP knowledge in the general population, measured through a demographic survey, was low (n = 125/8,414, 1.49% had heard of the drug). Medicalized delivery pathways created hostility to AGYW PrEP use. Key informants had higher levels of knowledge about PrEP and saw it as a needed intervention. Community norms around adolescent sexuality, which painted sexually active youth as irresponsible and disengaged from their own health, made many ambivalent towards a PrEP rollout to AGYW. Health care workers discussed ways to shame AGYW if they tried to access PrEP as they feared the drug would encourage promiscuity and “risky” behaviour. Others interviewed opposed provision on the basis of health care equity and feared PrEP would divert both drug and human resources from treatment programs.

**Conclusions:**

The health system in this poor, high-HIV incidence area had multiple barriers to a PrEP rollout to AGYW. Norms around adolescent sexuality and gatekeeper concerns that PrEP could divert health resources from treatment to prevention could create barriers to PrEP roll-out in this setting. Alternate modes of delivery, particularly those which are youth-led and demedicalize PrEP, must be explored.

## Introduction

Adolescent girls and young women (AGYW) (ages 15–24) remain disproportionately affected by HIV, comprising 11% of the global population and 20% of new HIV diagnoses [1]. In sub-Saharan Africa (SSA), where 80% of AGYW living with HIV reside, AGYW become infected five to seven years earlier than their male counterparts [2, 3]. This increased vulnerability partially stems from sociodemographic and behavioural characteristics. Many AGYW in SSA are constrained in their ability to bargain for existing safer sex tools (i.e. condoms) given gendered cultural norms around sexual activity [4, 5]. Low socio-economic status and diminished employment prospects incentivize transactional sex, further diminishing AGYW’s bargaining capacities in sexual relationships [6, 7].

Oral pre-exposure prophylaxis (PrEP) could help bridge the gender gap in prevention by offering AGYW an HIV prevention option they can more directly control than condoms. Daily oral regimens of antiretroviral drugs (ARVs) have proven effective at reducing the risk of HIV infection [8]. The World Health Organization (WHO) recommends PrEP be provided as part of a package of combination prevention options for groups at “substantial risk” of HIV (HIV incidence of approximately 3 per 100 person years)[9]. The South African National Department of Health supports PrEP as part of combination prevention strategies [10] and the country began a targeted rollout to female sex workers (FSWs) in June 2016 [11]. Efforts to make PrEP available to AGYW are underway [12].

Despite clinical effectiveness and policy maker enthusiasm, multiple randomized control trials of PrEP in AGYW in SSA have demonstrated low adherence to the drug and corresponding low efficacy [13, 14]. Open Label Extension trials (OLEs) in this population have shown higher levels of adherence than corresponding Randomised Controlled Trials (RCTs) [15]. While a PrEP rollout to AGYW in SSA is possible in well-resourced settings, broader support for potential and actual PrEP users and coordination of PrEP dissemination with existing clinical services will be critical [3, 16, 17].

Describing user, provider, and community views on and awareness of PrEP can help optimize PrEP provision to AGYW and facilitate scale-up by integrating new programs and tools with existing opportunities [14, 18–20]. Understanding such views and who holds them allows future PrEP provision programs to adapt to on-the-ground realities, minimize friction with community beliefs and practices, and build support with potential gatekeepers to the drug. For example, achieving buy-in for AGYW PrEP provision from health care workers and other opinion leaders will be critical, given the biomedicalization of HIV prevention [21]. The shift in emphasis from controlling behaviours (i.e. using condoms) to controlling bodies (i.e. supressing viral loads, using PrEP) means medical practitioners have the authority to decide which bodies are at significant enough risk to “deserve” care. These practitioners then continue to exercise authority by monitoring those receiving care. Such practices implicate previous social norms and relationships between providers and potential patients as critical facets of PrEP rollout [22, 23]. Biomedicalization also cedes substantial control to non-medical opinion leaders who might refer potential patients to providers.

Assessing current PrEP knowledge among community leaders, understanding their attitudes toward sexual and reproductive health services (SRHS) generally, and mapping ways to better target and retain potential users will be critical for a successful rollout in the north-east KwaZulu-Natal (KZN), a high HIV incidence area in rural South Africa. Unfortunately, little is known about awareness of or attitudes toward PrEP in this area, especially among those who may provide it. Better understanding community leaders’ narratives about SRHS will aid future PrEP provision by showing how to reduce delivery barriers and increase willingness to prescribe and support adherence to the drug.

*The aim of this* exploratory study was to understand the sexual health landscape for AGYW within which PrEP would be introduced in order to inform PrEP programming for AGYW in rural KZN. Specifically, we describe awareness and uptake of PrEP within the wider community and explore attitudes towards AGYW accessing PrEP (in theory) and general SRHS (in current practice) amongst potential gatekeepers to the drug. We use this to elucidate potential challenges or facilitators to PrEP access among AGYW.

## Methods

### Study design

This study was embedded within an implementation evaluation of the DREAMS Partnership in a rural, impoverished district in the north-east of KZN, South Africa. The area has an HIV incidence of > 5% per annum for AGYW. During 2017, when data collection for this study occurred, PrEP had only been provided in this area to FSWs through a single community-based organization funded by the DREAMS Partnership [24]. The DREAMS Partnership funded other evidence-based practices targeted at the general population with the goal of decreasing rates of HIV in AGYW (e.g. circumcision for male partners, condom distribution, sexual health education, cash grants to AGYW to continue school). Additional information about the DREAMS Partnership and the implementation evaluation at this field site has been described in other publications [24, 25].

Throughout 2017, we used a mixture of demographic surveillance and key informant interviews to quantify the awareness and uptake of PrEP at the population level and describe the attitudes of potential gatekeepers to PrEP. Multiple data types were used to better contextualize the quantitative data as well as understand the environment in which future PrEP rollouts could occur, thus enabling barriers and facilitators to PrEP for AGYW to be identified.

### Setting and population

The study area, a predominately rural region with high rates of poverty and unemployment, is in the Hlabisa sub-district of the uMkhanyakude district in KZN [26]. HIV rates are persistently high with an antenatal prevalence of more than 40% [27]. The area hosts the Africa Health Research Institute (AHRI) (formally the Africa Centre for Health and Population Studies) which, since 2000, has used household-based surveys to collect demographic data from a population of approximately 100,000 individuals living in an area of 438 km^2^ within the district. AHRI surveys households three times per year to collect information on births, deaths, and migration patterns for all household members, including non-residents. Resident household members who were 15 years or older were invited to participate in annual, face-to-face, individual surveys. For the duration of the DREAMS Partnership implementation evaluation, the Bill and Melinda Gates Foundation provided additional funding which allowed for the addition of a DREAMS-specific interview module to the demographic survey. This module measured PrEP awareness and uptake at a population level [25]. There was no PrEP provision in this area until 2016, when PrEP was rolled out to FSWs through the DREAMS Partnership.

### Qualitative study

A purposive sample of 52 potential gatekeepers was assembled, including 10 people affiliated with the local Department of Education (DoE), 24 people affiliated with the local Department of Health (DoH), ten DREAMS stakeholders, four DREAMS evaluation fieldworkers, and four people affiliated with a local non-governmental organization providing PrEP to FSWs. Semi-structured, in-depth interviews were conducted from May to November 2017. Interviews lasted an hour on average and explored perceptions of the HIV epidemic in the area and its relation to adolescents, current awareness of PrEP and other novel biomedical HIV prevention tools, and attitudes toward PrEP provision, especially among AGYW.

Most interviews were conducted in isiZulu by research assistants (five females and four males) who had lived in the community for five to ten years and were familiar with community practices and norms. Fifteen of the DoH interviews and all field worker interviews were conducted by SEN, a female social scientist with prior PrEP research experience outside of South Africa. Interviews were recorded, transcribed verbatim, and translated to English if necessary. Interviewers also completed formal summary notes on the interviews which were shared among the research team to help shape future lines of inquiry. Interviews were coded iteratively. A unified codebook was created based on expected themes (from the interview guide and formal summary notes) and emerging ones. Study team members both coded the same scripts and discussed emerging themes at multiple points during the process to enhance validity. ATLAS.ti was used to store and manage the transcripts [28].

### Quantitative analysis

Data from the AHRI’s demographic surveillance platform were used to explore PrEP knowledge. Information on sampling has been described in other publications [26]. Data were collected by teams of two trained fieldworkers (age-sex matched where possible) visiting eligible individuals in their households. Fieldworkers conducted interviews with household members separately to maintain privacy and confidentiality. Population awareness and uptake of PrEP was quantified (mean and 95% confidence interval) using the following questions: “*Have you ever heard of PrEP? (These are tablets that people who do not have HIV can take to reduce the chances of catching HIV)*” and “*Are you currently taking PrEP to prevent HIV?*”.

To identify if there were any demographic details correlated with PrEP awareness and uptake, we stratified awareness and uptake by gender and age band. We also stratified by previous engagement with HIV testing (measured through the question “*Have you ever received a test result for HIV?*). For female respondents only, we stratified by current and previous engagement with contraceptive services (measured through the questions “*Are you currently using any contraceptive methods to prevent pregnancy?*” and “*In the past 12 months, have you used any method to avoid getting pregnant?*”). Data cleaning, tabulation, and confidence interval calculations were performed using Stata version 15 [29]. We did not perform any further tests for significance as there were low levels of awareness and uptake, and given the large sample size, there was a risk of finding spuriously significant associations [30].

### Reflexivity statement

SEN is a white, North American researcher with past work disseminating PrEP to vulnerable populations in the US. The rest of the study team had prior experience working in the study area including MS, an HIV physician who has lived and worked with vulnerable populations in India, Burma, and South Africa, and JS, an anthropologist with extensive experience living and working in southern and eastern Africa. AHRI’s multi-decade engagement in this study area as well as the diverse perspectives of the study team strengthened study design, data collection and analysis.

## Findings

### Population awareness and uptake of PrEP

Of 13,562 eligible participants, 62.1% (n = 8,414) participated in the general health survey which contained questions related to PrEP. Within the subset of participants who answered the general health survey, 1.49% had heard of the drug (n = 125, 95% CI  1.23–1.74), Only one person, a female between the ages of 25 and 34, reported using PrEP. Table 1 lists demographic details and PrEP awareness stratified by gender, age, history of HIV testing and current/previous contraceptive use.

**Table 1:** Demographic characteristics

Variable	N (%)	PrEP awareness (N =)	PrEP awareness % (95% CI)
Overall	8,414 (100%)	125	1.49 (1.23–1.74)
Female	6,129 (72.84%)	101	1.65 (1.33–1.97)
Male	2,285 (27.16%)	24	1.05 (0.63–1.47)
Age 15–19	1,523 (18.1%)	13	0.85 (0.39–1.32)
20–24	1,045 (12.42%)	15	1.44 (0.71–2.16)
25–34	1,648 (19.59%)	32	1.94 (1.28–2.61)
35–44	1,117 (13.28%)	36	3.22 (2.19–4.26)
Over 45	3,081(36.62%)	29	0.94 (0.60–1.28)
Current contraceptive use (female only)	1,048 (45.82%)	32	3.05 (2.01–4.10)
Past contraceptive use (female only)	1,023 (39.56%)	31	3.03 (1.98–4.08)
Previously tested for HIV	6,351 (76.11%)	106	1.67 (1.35–1.98)

**Fig. 1:**
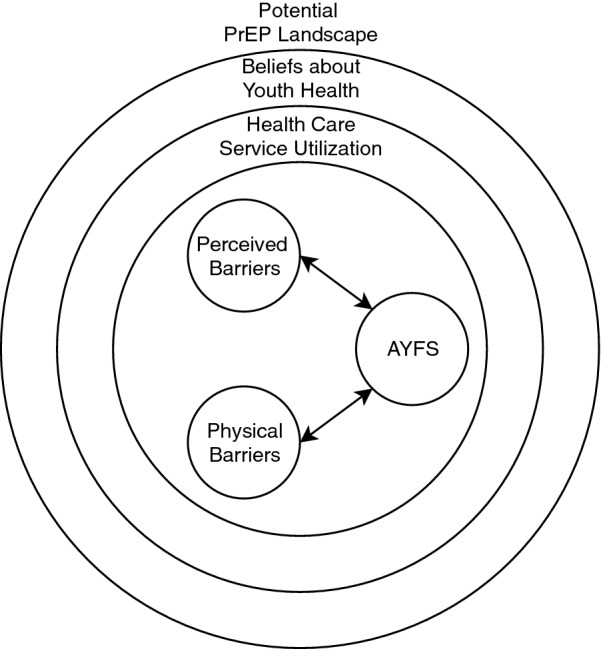
Interactions among emerging themes

Several themes emerged during the interviews to describe the reception of this area to AGYW PrEP rollouts. Figure 1 shows the interaction among these themes. Discussions with potential PrEP gatekeepers demonstrated that future provision will likely be shaped by multiple factors. These factors include.

- Existing beliefs about how youth choose to access services and which services providers believe they should be accessing,
- Existing barriers to youth accessing SRHS, and by
- Adolescent and youth friendly services (AYFS) that are currently being implemented at clinics.

### Potential PrEP landscape

Participants were unaware of PrEP, or, if they had heard of it, were sceptical of its efficacy. For example, in discussions they shared “not knowing”, “not having an idea” about what PrEP was, and asked if PrEP “really worked”.

In contrast, local DoH employees had often heard of the pill through professional engagements and were more likely to trust its efficacy.

Once introduced to PrEP, many participants welcomed the availability of a new HIV prevention tool on principle. They couched this acceptance in terms of wider community needs, usually appealing to high local rates of HIV. However, they also expressed significant concerns about introducing PrEP to AGYW. Participants believed that providing PrEP to adolescents would divorce the fear of HIV from sex and discourage condom use while encouraging sexual behaviour. As one DoH employee explained, *“[giving adolescents PrEP] will mean setting them free to engage in unprotected sexual intercourse.*”

Generally, participants did not name possible barriers to a PrEP rollout. The few they named matched problems that have characterized clinical trials of PrEP in AGYW (i.e. adolescents would not be able to remember to take a pill every day). Some worried clinic environments would discourage adolescents from seeking services, both because nurses would scold adolescents who sought SRHS and because wait times for services would be substantial. Few mentioned potential PrEP facilitators, but some referenced the possibility of using peer educators, providing adherence counselling, and dispensing PrEP at non-traditional sites, such as in schools and through mobile clinics. Others believed that nurses would be eager to help with a rollout to adolescents, given that it provided a way for them to protect “*the future*” of the country.

### Beliefs about youth health

Participants often discussed their beliefs about adolescent (often AGYW-specific) health beyond PrEP. Three major themes arose inductively from these reflections: moral anxiety about youth sexuality, guardianship, and concerns about equity.

#### Moral anxiety about youth sexuality

Participants often expressed significant concerns about the sexual health of the adolescents with whom they worked. While adolescent HIV infections worried many, participants spent more time discussing their fears about the high rates of other STIs and pregnancy. Elevated rates were attributed to a variety of influences, including lack of condom use, disinterest in seeking treatment, high substance abuse rates, and transactional sex relationships. Participants expressed a moral anxiety around a perceived existential crisis of adolescents’ “*reckless”* behaviour. From a field interviewer’s notes on an interview,

“[A DoH employee] said that the reason why young people are at risk is because they are attracted to things; they fail to learn from their mistakes.”

This judgement frequently emerged when discussing transactional sex relationships. Participants rarely discussed power imbalances within these relationships or the reliance on such relationships for economic survival and instead attributed this behaviour to AGYW’s desire for *“nice stuff.”* Participants often advocated for abstinence-only education, rationalizing that once youth became sexually active, they became impossible to control. As a DoE employee explained,

With regards to the issue of dating, [teachers] would say that [children] are now fine as they are now not dating anyone… [When the child stops dating], you see that the child is now a child, not that animal she has been, being uncontrollable.

#### Guardianship over adolescent sexuality

Given concerns for how youth conducted themselves and the perceived consequences of this conduct, participants, especially DoE and DoH employees, saw youth as unable to determine their own health needs. As such, these participants believed that they (or their colleagues) were responsible for making sure that youth did the “*right things*.”

Generally, participants began by expressing how adolescents were disengaged (either though lack of knowledge or lack of care) from their own health. As one district stakeholder explained:

“Young people are special, and they don’t understand other things well. They fail to take good decisions towards other things.”

A nurse put it more bluntly:

“[Young women] have this ‘not care- don’t care’ attitude. Like, ‘I don’t care what happens to me tomorrow.’”

To mitigate this perceived disinterest, participants described how they would make decisions on behalf of the adolescents with whom they interacted. Often, this took the form of performing unrequested STI/pregnancy tests when AGYW sought medical care. They also described how they or their colleagues strongly advised and even scolded AGYW who sought SRHS in the hopes that they could curb sexual behaviour. As a stakeholder explained:

“…nurses act as parents and if ever they are coming to just, you know, ask for contraceptives the nurse will just act as, you know, a guardian and tell the child that, ‘Why are you coming because you are not young, you’re not supposed to do this, you’re not supposed to take contraceptives,’ you know all those things.”

This intergenerational power dynamic may provide some benefit for an AGYW PrEP rollout. Some participants referenced the need to protect young women from HIV, given that they were the “*future*” of the country, as a motivator for becoming involved in PrEP work. The only nurse currently working on a PrEP rollout expressed this emotional draw to PrEP work, stating:

“… when I heard … about [PrEP], I said, ‘Let me go take a chance and see what happens because I want to see our community living.’ And when I heard them speaking about having treatment on trial to see if it can help prevent the community from being infected, I said, ‘No, I will not be left behind. Let me be one of the pioneers of that, so that my heart will be at peace and happy when I go home to retire, as I would have witnessed the job done.’”.

#### Concerns about equity

Some of the participants who were less stigmatizing of adolescents seeking SRHS noted the perceived unfairness (and even short-sightedness) of focusing only on AGYW. This was especially true when participants discussed the DREAMS Partnership. The DREAMS Partnership provided the bulk of its services to AGYW, which participants believed alienated young men. Participants often noted that they were just as, if not more, concerned about the sexual health of adolescent boys and young men (ABYM) and similar sexual health resources, including PrEP, should be offered to ABYM as well. As a DoE employee explained,

“You cannot fence your plants, but not cuff your goats. … What I mean is that DREAMS concentrates in girls and boys are not given that attention. They even ask me why they are not involved. Instead the attention is payed more to girls only. Girls do not get themselves pregnant, but a boy makes a girl. So, I think paying attention to girls is of much help, but they abandon boys who have an ability to impregnate.”

Participants also worried about the “fairness “of providing PrEP generally. Many believed that the government lacked the resources to conduct such a rollout and worried that there would be a future time during which they would have to choose between providing ARVs for prevention (through PrEP campaigns) and providing them for treatment (especially through the new guidelines that mandated starting ARVs when a patient first tested positive, regardless of CD4 count). Some DoH employees spoke of plans to ration PrEP to ensure they would always have enough ARVs for treatment. As one DoH employee explained,

“You look at the… drug stock outs, that we actually experience in our DoH clinics. The answer you’re going to get from a nurse in the clinic will be like, ‘What happens when I’m out of drugs? So, why must I be giving PrEP instead of treating- trying to prevent the disease propagation in someone who’s already HIV positive?’ So, they’re going to think of it that way.”

### Health care service utilization

Participants also described how adolescents interacted with health care services, generally noting that adolescents eschewed clinic visits when possible. Some described the steps clinics were taking to attract AGYW and adolescents.

#### Perceived barriers

Participants cited three reasons for believing that adolescents viewed clinics as places of stigma. First, DoH workers admitted that they or those they knew sought to dissuade adolescents (especially AGYW) from accessing SRHS. Some interviewed believed that through nagging and scolding they could dissuade their patients from sexual activity. Participants acknowledged this behaviour often drove potential patients away. Second, participants believed adolescents avoided clinics for fear they would be seen there by their family or other community members and face reprimands. As a DoE employee explained,

“Most [pregnant young women] come to the clinic when they are at an advanced stage. … They hide the pregnancy because of fear, and they tie the stomach with a belt. Their fear is that they will be seen at the clinic standing in a queue waiting for an ultrasound, when they have not reported to their parents that they are pregnant.”

Third, participants sometimes discussed the district policy of sequestering HIV testing and treatment away from the main clinic in mobile trailers (“*park homes*”). This policy aimed to shorten waiting times for specific patients but, given the existing local stigma against people living with HIV, instead discouraged adolescents from seeking HIV tests for fear that others would assume they were HIV positive. As a field worker explained,

“So now people will know that this section with park homes is for- it’s basically dealing with HIV related issues and then when you go to the main building, it’s for other, like for minor ailments and other diseases but when you go to park homes, it’s basically for HIV related issues. Now people are afraid of going to the clinic. Yeah. Because the setting is stigmatized.”

#### Physical barriers

Participants acknowledged that they often did not have the resources to attract adolescents. Understaffing resulted in long lines that discouraged people from seeking care. The wait times were especially problematic for school-aged adolescents who did not have the ability to wait at the clinic for long periods of time. Participants also bemoaned the lack of funding to invest in more mobile and school-based outreach, which they believed would help them reach young people. As a DoH employee complained,

“So, there’s lack of stuff, there’s no stuff. Even the infrastructure at the clinics is not conducive enough to accommodate this youth friendly service, because you find that the clinic is, is maybe- maybe some of the clinics is having two consultation rooms. You can’t have now one that is now just going to be designated for the youth. … There are no resources, there is shortage of staff, the infrastructure is not conducive. It’s just a pain, coming to work”.

#### Adolescent and Youth Friendly Services (AYFS)

In recognition of the barriers that adolescents generally faced when seeking care, many participants spoke about the new, district-wide push for AYFS. Participants explained that guidance from the local DoH recommended clinics dedicate a separate room in which adolescents could wait for services while watching TV, reading magazines, or otherwise entertaining themselves. Clinics should also set up a “*happy hour*” time after school during which adolescents could be seen without delay and train at least one nurse to be a “*youth friendly champion*.” This nurse would ensure an adolescent-friendly, non-judgmental environment. Participants hoped these services would build rapport between clinics and adolescents so that adolescents would feel comfortable seeking both health knowledge and treatment from clinics.

Participants noted that multiple barriers existed to implementing AYFS. Limited resources prevented allocation of youth-dedicated spaces or staff to run the “*happy hour*.” Frequent staff turnover often left the role of “*youth friendly champion*” vacant. The minimal training for people who rotated through the position failed to change a culture of judgement against youth sexual activity. A DoH employee lamented the failed promise of AYFS by saying,

“There are other programmes that we implement at our clinics, like youth friendly, where we encourage the youth to come to the facilities even if they are not sick, just for information. Unfortunately, that project does not want to be fully operational.”

## Discussion

In this rural setting with high rates of poverty and HIV incidence, we found that there were multiple barriers within the health system to a PrEP rollout to AGYW, exacerbated by health care providers lack of preparedness and existing norms around adolescent sexuality. While PrEP and other SRHS for AGYW ( e.g. long acting contraceptives) have been marketed as ways of increasing empowerment and autonomy in young women, the biomedicalization of these services and their delivery removes power from the user and shifts it to the provider [31]. We found that within current delivery models, PrEP will remain embedded within existing biases and generational power relations, both real and perceived, which will harm a rollout through the health system. Community norms around adolescent sexuality expressed by potential PrEP gatekeepers showed that while many agreed an intervention like PrEP was needed in the abstract, they would be unwilling to facilitate AGYW PrEP access. This finding was especially pronounced among DoH staff who saw themselves as guardians of youth health and, more broadly, as arbiters of equitable access to healthcare. Many were (or believed their colleagues would be) reluctant to prescribe PrEP to AGYW for fear it would increase sexually risky behaviour and divert drugs from those living with HIV.

The lack of population-level PrEP awareness will likely heighten the gatekeeping dynamic between potential users and community leaders, particularly during the initial general PrEP rollout. DoH employees, especially, could have outsize power in choosing to whom they recommend the drug and whom they discourage from using it, especially when low-levels of population knowledge prevent potential users from self-advocating and voicing the reasons why they would like to access PrEP [32, 33]. Similar gatekeeping dynamics predicated on community norms around adolescent sexuality have been seen in past SRHS campaigns that used medicalized delivery pathways. In these situations, DoH employees who worried certain medical interventions would encourage sexual activity in AGYW (e.g. contraception, termination of pregnancy services) succeeded in hindering and derailing previous rollouts [34–36]. Any PrEP rollout to AGYW in this area will benefit from substantial efforts to address existing norms around adolescent sexuality. Future research to develop and pilot PrEP training for gatekeepers to the drug that combats stated biases against SRHS provision to AGYW could be especially helpful.

We found that providing AYFS at clinics does not seems to be shifting norms around adolescent sexuality. While some interviewed confirmed that many local clinics met standards for AYFS accreditation, judgement of adolescents accessing SRHS remained a major barrier to care. While this may have been the result of limited funding and high rates of staff turnover, our finding matches earlier research showing that even when clinics can meet appropriate benchmarks for AYFS [37, 38], youth perceptions of care friendliness barely improve [39]. This suggests that the current structure of AYFS is inadequate for creating a welcoming, non-judgemental environment. Should PrEP be provided through clinics, the clinics in this area, (including those with existing AYFS) will require substantial changes in staffing and training to attract AGYW for PrEP.

Our findings offer a potential explanation for the failure of AYFS to change norms around adolescent sexuality. Medicalized delivery pathways, even those engineered for adolescents, reinforce existing power relationships and prioritize the intergenerational anxieties DoH employees have about adolescent sexuality. DoH employees saw their career as a means of guardianship for youth’s future health. Those interviewed believed that they and their colleagues had a moral responsibility to protect young women from engaging in sexual behaviour and thus a moral imperative to deny them PrEP because they feared prescribing PrEP would increase sexual activity, decrease condom use, and lead to more pregnancies and STIs. This framing of service denial as a moral issue by nurses in South Africa has been shown in relation to contraceptive services [35, 36, 40] but our research is some of the first in this area to expand this framing to PrEP and HIV prevention outside of condoms.

DoH employees’ view that their position had moral stakes extended beyond adolescent guardianship. DoH employees believed they had a moral imperative to ensure equitable access to health care. The introduction of a new drug in a health care setting those interviewed believed to be resource impoverished worried many. Some DoH employees began to categorize those who might seek ARVs as either deserving (those who were using them for treatment) or undeserving (those who were using them for prevention). While the division of “deserving v. undeserving” [41] has been explored in gatekeeping dynamics, it has been rarely explored in the context of treatment and prevention. Previous work, in contrast, has shown that in past drug rationing, South African nurses were more likely to see people living with HIV as undeserving of care [20].

While some interviewed demonstrated that the moral imperatives by which DoH employees characterised their work could be used as an entry point to PrEP provision (i.e. through impressing upon HCWs that PrEP was a way to protect AGYW), it was not clear that this would work for a significant number of potential PrEP providers. Additionally, medicalized pathways, even those which profess AGYW empowerment, defer to medical providers and institutions that have been hostile to SRHS provision to AGYW. Future studies should explore the potential of demedicalizing PrEP and of using alternate modes of community-led or even youth-led delivery [42]. Such modes of delivery may help increase access and adherence to PrEP [43, 44]. They may also create new norms around adolescent sexuality and better combat stigma than current modes of delivery [45, 46].

## Limitations

While our study contributes important findings to support South African efforts to provide PrEP to AGYW, we acknowledge its limitations. All interviews and survey responses were collected in a rural area of KZN. The results may not be generalizable to other areas of South Africa even though they provide important contextual information about similar areas with high HIV incidence. The quantitative data that measured the current population awareness of oral PrEP was collected as part of a multi-hour longitudinal survey. Survey fatigue may have incorrectly skewed answers. The incredibly low level of PrEP knowledge still suggests that even with some skew, consciousness raising around PrEP will be essential in this area. Qualitative interviews were conducted before PrEP was available for any segment of the general population. The opinions respondents stated were based on an abstract idea of what oral PrEP may do when introduced to the community. These opinions may change in a general population PrEP rollout. Opinions may have also changed after the interview: for some participants, this was their first introduction to the idea of ARV-based HIV prophylaxis and their views could have evolved with further reflection. Finally, respondents may have believed they needed to profess more excitement about PrEP and/or hide concerns about the drug or its potential rollout to please the interviewer(s). Given the range of opinions; however, significant discordance between their personal beliefs and those professed during interviews is unlikely.

## Conclusion

The health care system in this area of rural South Africa was unprepared to support a rollout of PrEP to AGYW. Community norms around adolescent sexuality, clinic environments that were ambivalent to AGYW seeking SRHS, and the moral conviction of some DoH employees who believed that PrEP provision would harm community members have the potential to adversely affect a PrEP rollout to AGYW. With current modes of delivery, PrEP rollouts may not offer the empowerment and sexual autonomy advertised for young women. Future research must investigate alternate modes of delivery, especially those that demedicalize PrEP and provide more opportunities for community and youth leadership in its delivery.

## Data Availability

The datasets generated and analysed during the current study are available in the AHRI Data Repository, which can be found at data.africacentre.ac.za. The qualitative manuscripts analysed during the current study are not publicly available due to privacy concerns of those interviewed but are available from the corresponding author on reasonable request.

## Abbreviations

ABYM: Adolescent Boys and Young Men
AGYW: Adolescent Girls and Young Women
AHRI: Africa Health Research Institute
ARV: Anti-Retroviral
AYFS: Adolescent and Youth Friendly Services
DoE: Department of Education
DoH: Department of Health
FSW: Female Sex Worker
HCW: Health Care Worker
KZN: KwaZulu Natal
OLE: Open Label Extension
PEPFAR: President’s Emergency Plan for AIDS Relief
PrEP: Oral Pre-Exposure Prophylaxis
RCT: Randomized Control Trial
SSA: Sub-Saharan Africa
SRHS: Sexual and Reproductive Health Services
WHO: World Health Organization
